# Assessment of Water Quality in Asa River (Nigeria) and Its Indigenous *Clarias gariepinus* Fish

**DOI:** 10.3390/ijerph8114332

**Published:** 2011-11-18

**Authors:** Olatunji M. Kolawole, Kolawole T. Ajayi, Albert B. Olayemi, Anthony I. Okoh

**Affiliations:** 1 Applied and Environmental Microbiology Research Group, Department of Biochemistry and Microbiology, University of Fort Hare, Private Bag X1314, King William’s Town Road, Alice 5700, South Africa; E-Mail: aokoh@ufh.ac.za; 2 Infectious Diseases and Environmental Health Research Group, Department of Microbiology, University of Ilorin, P.M.B. 1515, Ilorin, Nigeria; E-Mails: kolatemaj@yahoo.com (K.T.A.); bolayemi_2005@yahoo.com (A.B.O.)

**Keywords:** Asa River, physicochemical parameters, histological, water quality, pollution

## Abstract

Water is a valued natural resource for the existence of all living organisms. Management of the quality of this precious resource is, therefore, of special importance. In this study river water samples were collected and analysed for physicochemical and bacteriological evaluation of pollution in the Unity Road stream segment of Asa River in Ilorin, Nigeria. Juvenile samples of *Clarias gariepinus* fish were also collected from the experimental Asa River and from the control Asa Dam water and were analysed for comparative histological investigations and bacterial density in the liver and intestine in order to evaluate the impact of pollution on the aquatic biota. The water pH was found to range from 6.32 to 6.43 with a mean temperature range of 24.3 to 25.8 °C. Other physicochemical parameters monitored including total suspended solids, total dissolved solids, biochemical oxygen demand and chemical oxygen demand values exceeded the recommended level for surface water quality. Results of bacteriological analyses including total heterotrophic count, total coliform and thermotolerant coliform counts revealed a high level of faecal pollution of the river. Histological investigations revealed no significant alterations in tissue structure, but a notable comparative distinction of higher bacterial density in the intestine and liver tissues of *Clarias gariepinus* from Asa River than in those collected from the control. It was inferred that the downstream Asa River is polluted and its aquatic biota is bacteriologically contaminated and unsafe for human and animal consumption.

## 1. Introduction

Water is vital to the existence of all living organisms, but this valued resource is increasingly being threatened as human populations grow and demand more water of high quality for domestic purposes and economic activities [1]. The quality of any body of surface or ground water is a function of either or both natural influences and human activities [2,3]. It is now generally accepted that aquatic environments cannot be perceived simply as holding tanks that supply water for human activities. Rather, these environments are complex matrices that require careful use to ensure sustainable ecosystem functioning well into the future [1].

Rivers are the most important freshwater resource for man. Unfortunately, river waters are being polluted by indiscriminate disposal of sewerage, industrial waste and plethora of human activities, which affects their physico-chemical characteristics and microbiological quality [4]. Pollution of the aquatic environment is a serious and growing problem. Increasing numbers and amounts of industrial, agricultural and commercial chemicals discharged into the aquatic environment have led to various deleterious effects on aquatic organisms. Aquatic organisms, including fish, accumulate pollutants directly from contaminated water and indirectly *via* the food chain [5,6].

Owing to the large quantity of effluent discharged to the receiving waters, the natural processes of pathogen reduction are inadequate for protection of public health. In addition, industrial wastes that alter the water pH and provide excessive bacterial nutrients often compromise the ability of natural processes to inactivate and destroy pathogens [7]. The extent of discharge of domestic and industrial effluents is such that rivers receiving untreated effluent cannot provide the dilution necessary for their survival as good quality water sources. The transfer of unfavourable releases from industries is detrimental to human and animal health and safety [8]. Disposal of sewage wastes into a large volume of water could increase the biological oxygen demands to such a high level that all the available oxygen may be removed, consequently causing the death of all aerobic species, e.g., fish [9].

Prevention of river pollution requires effective monitoring of physico-chemical and microbiological parameters [10]. In most countries, the principal risks to human health associated with the consumption of polluted water are microbiological in nature [11].The bacteriological examination of water has a special significance in pollution studies, as it is a direct measurement of deleterious effect of pollution on human health [12]. Coliforms are the major microbial indicator of monitoring water quality [13,14]. The detection of *Escherichia coli* provides definite evidence of faecal pollution; in practice, the detection of thermotolerant (faecal) coliform bacteria is an acceptable alternative [11].

Histopathological alterations can also be used as indicators for the effects of various anthropogenic pollutants on aquatic biota and are a reflection of the overall health of the entire population in the ecosystem [6]. Histopathological changes have been widely used as biomarkers in the evaluation of the health of fish exposed to contaminants, both in the laboratory [15–17] and field studies [18–20].

Asa River is a major river of economic, agricultural and environmental significance in Ilorin—the capital city of Kwara State, Nigeria. The river receives effluents from industries located along its course, apart from domestic wastes and other activities carried out along it that contribute to its pollution. It was also reported that the major identified source of pollution of Asa River was direct runoff of effluents from the industries [8]. Moreover, it was pointed out that the river is subject to high level of eutrophication due to the organic matter and industrial effluents discharged into it [21]. This informed the need to evaluate the impact of pollution on the river segment and its aquatic biota. A study of this kind has not been carried out on the river.

Our group aimed to assess the ecological and public-health aspects of pollution impact on the experimental Asa River segment by determining the physicochemical parameters, bacteriological river water quality indicators and histological investigations for possible pathologic effects and bacterial density in the viscera of indigenous samples of catfish (*Clarias gariepinus*), a commercially important fish. *Clarias gariepinus* is common in Nigerian rivers, including Asa River, and it is consumed by many in Ilorin city.

## 2. Materials and Methods

### 2.1. Study Area

**Figure 1:**
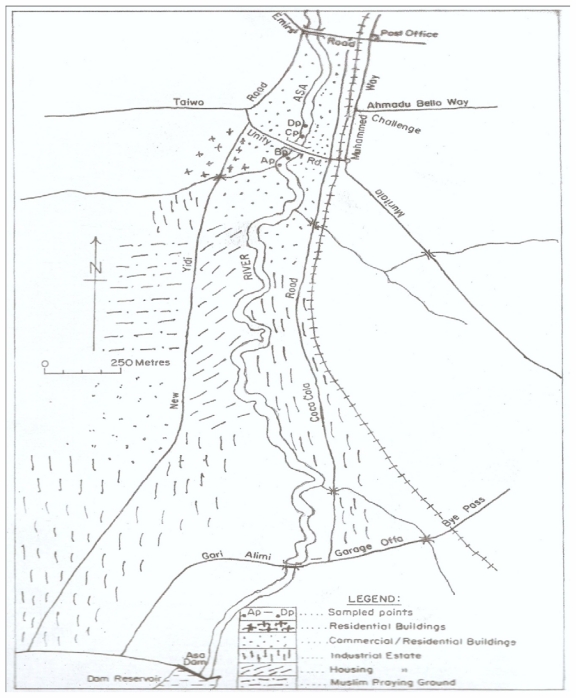
A geographical map of the Asa River segment showing the study area and sampling points.

The study was conducted on the Asa River segment at the exit of the industrial estate around Unity Road, Ilorin, North Central Nigeria (8°28′N, 4°38′E to 8°31′N, 4°40′E). Asa River is the major water body in Ilorin, its course enters the southern end of the industrial estate from Asa Dam and it runs northwards through residential and commercial areas of Ilorin city. A scaled geographical map of the course of Asa River showing the study area and sampling points is shown in Figure 1. Apart from the containment of industrial effluents from several manufacturing plants within the estate, the river also serves as a recipient of domestic (sewage) wastes and agricultural waste run offs along the bank of the river. Along the area of the river segment are shopping complexes, a hospital, banks, a car park and a mini market for the sale of fresh vegetables and fish.

### 2.2. Water Sampling

Grab samples of water were taken from four different points—Ap, Bp, Cp and Dp—on the Asa River segment at the Unity Road bridge in Ilorin: points Ap and Bp are located at one side of the bridge and points Cp and Dp are at the other side of the bridge of the stream flowing northwards. Water sampling was carried out at weekly intervals for a period of 5 weeks between September and October, 2009. Samples were collected following the standard sampling guidelines and methods [11]. The samples were taken into pre-sterilized bottles kept in ice-boxes and transported immediately to the laboratory for physicochemical and bacteriological analyses.

### 2.3. Fish Sampling and Tissue Preparation

Juvenile fish samples (100) of *Clarias gariepinus* (African catfish) were collected from the river section by the aid of a fisherman using conventional fish net traps in the early hours of the day. Samples of the same fish species were also collected from the Asa Dam reservoir water, a pollution-restricted reservoir, which was to serve as comparative control water during histological analysis. After dissection of fish samples in the laboratory, parts of the liver and intestine were carefully removed and prepared for histological studies [6].

### 2.4. Physico-Chemical Analysis

The collected river water samples were analysed in the laboratory for pH, temperature, total dissolved solids (TDS), total suspended solids (TSS), biochemical oxygen demand (BOD) and chemical oxygen demand (COD) per standard methods [12,22]. The pH of the samples was determined using a sensitive digital-electrode pH meter (Metrohm 632). The temperature of each sample collected was measured on-site with the use of a mercury bulb thermometer. The TDS and TSS of water samples were determined by the filtration and evaporation method while BOD and COD were determined using the alkaline-azide modification of Wrinkler’s titration method [22].

### 2.5. Bacteriological Analysis

Quantitative bacteriological analysis of the water samples were carried out by using standard plate count (SPC) to enumerate total heterotrophic bacteria (TH) and using the membrane filtration (MF) method to enumerate total coliforms (TC) and total thermotolerant coliforms (TTC) [11,12]. Total coliforms and total thermotolerant coliforms were detected and quantified with the use of Eosin methylene blue (EMB) agar and their incubation at 37 °C and 44.5 °C respectively. Their counts were expressed in cfu/100mL of the water. The IMViC and other biochemical tests including production of greenish metallic sheen on the EMB agar plates were used to confirm and identify *Escherichia coli* [23,24].

### 2.6. Histological Investigations

Specimens prepared for histological examination are from liver and intestine of juvenile *Clarias gariepinus* fish samples collected from both the test Asa River segment and the control Asa Dam water. The histological examination was done using the standard tissue examination techniques [6,23,25].

#### 2.6.1. Examination for Alteration in General Tissue Structure

Fixation of the specimens (tissues of liver and intestine) was done in 10% neutral-buffered formalin for 72 hours. After fixing, the tissues were dehydrated by treating with ascending grades of alcohol solutions (70% to absolute). The tissues were then cleared in xylene, impregnated with molten paraffin wax and embedded in paraffin wax using embedding moulds which confer rigidity to the pieces of tissue for easy cutting of sections [6,25].

#### 2.6.2. Sectioning

Sections were cut with the use of Leica Rotary Microtome to section thickness of 4 μm from the paraffin wax blocks. The cut sections were placed onto 20% alcohol on a large slide, from where they were gently floated on water bath preheated to about 45 °C, after which they were picked from water and mounted on clean grease-free microscope slides. Slides with sections were dried for about 30 minutes before staining.

#### 2.6.3. Haematoxylin and Eosin (H and E) Staining for General Tissue Structure

Haematoxylin and eosin staining were done as described elsewhere [25]. The cut-out sections were dewaxed in xylene and hydrated through descending grade of alcohol (absolute—80%–70% water). The slides were stained in Harris haematoxylin for 10 minutes and then rinsed in water. The slides were dipped in 1% HCl in 70% alcohol for 1 minute for differentiation and then rinsed in water. Blueing was done for 10 minutes in tap water after which counterstaining was done with 1% eosin for 1 minute. The slides were then rinsed in water, dehydrated, cleared and mounted for examination on the microscope followed by photomicrography.

#### 2.6.4. Examination of Bacterial Density in Tissues

Demonstration of bacteria in the fish tissues was done using special staining by gram’s staining modifications [23,25]. Thin sections of only the liver and intestine tissues of the fishes from both the test river water and control water were stained by special gram’s staining method for bacterial examination.

### 2.7. Statistical Analysis

The data obtained was subjected to descriptive statistics using Mean and Standard Error of mean. Also, analysis of variance (ANOVA) test under Completely Randomized Design (CRD) was carried out while Duncan Multiple Range Test (DMRT) was used to test for the means that are significantly different from the other, which are presented by alphabets in superscripts in the tables presented.

## 3. Results

**Figure 2:**
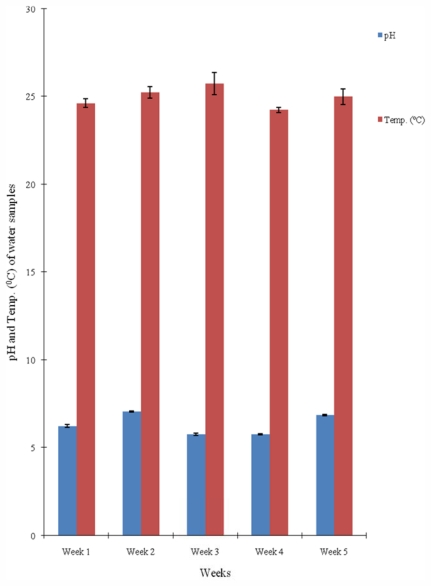
Temporal variations in pH and Temperature values of water samples from Asa river measured over a five-week period.

**Figure 3:**
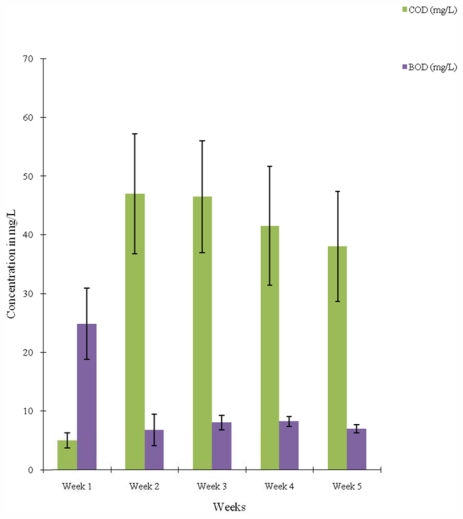
Temporal variations in COD and BOD contents of water samples from Asa river segment measured over a five-week period.

**Figure 4:**
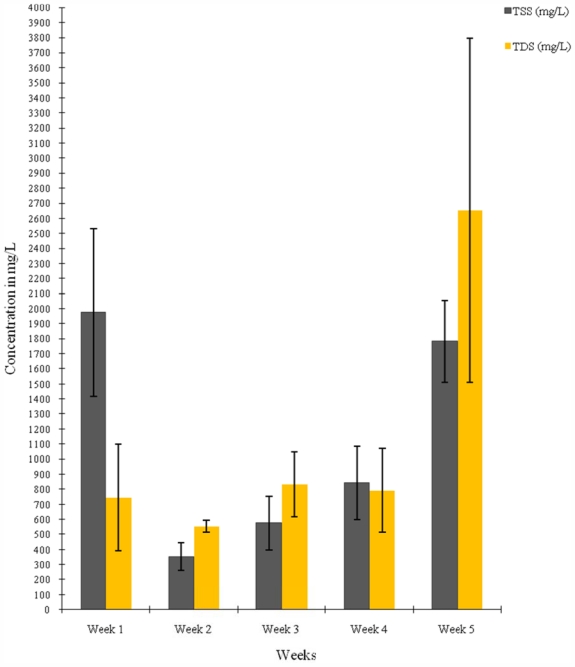
Temporal variations in TSS and TDS contents of water samples from Asa river measured over a five-week period.

The results of the mean values of the physicochemical parameters of the water samples from the four sampling points on the river segment are presented in Table 1 while Figures 2–4 display the variations in pH and temperature values; BOD and COD contents; and the TSS and TDS contents of the water samples, respectively, measured for a five-week period. The mean pH varied between 6.2 and 6.43 (Table 1 and Figure 2). The mean temperature of the water at different sampling points ranged from 24.5 °C to 25.3 °C (Table 1), while the temperature in weeks ranged from 24.3 °C to 25.8 °C (Figure 2). The mean values of the total suspended solids (TSS) and total dissolved solids (TDS) range from 766.60 to 1,498.60 mg/L and 704.60 to 1,799.80 mg/L respectively. The lowest values of both TSS and TDS were recorded at the sampling point Cp.

The mean values of the chemical oxygen demand (COD) oscillated between 23.20 mg/L to 58.40 mg/L and also between 5.00 mg/L and 46.50 mg/L for the samples taken along the sampling points and over the weeks respectively (Table 1 and Figure 3). The COD values were remarkably high across the weeks with the exception of week 1 (Figure 3). The mean BOD values ranged from 6.48 mg/L at point Dp to 14.00 mg/L at point Ap. There was a consistent decrease in the BOD values along the sampling points from Ap to Dp (Table 1). The BOD across the weeks was highest in week 1 and lowest in week 2 with the values of 24.85 mg/L and 6.80 mg/L respectively (Figure 3).

The mean total viable count of the total heterotrophic bacteria (TH) at 37 °C, total coliforms (TC) and total thermotolerant (faecal) coliforms (TTC) of the water samples from different sampling points are presented in Table 2. The mean TH, TC and TTC of the water samples of Asa River segment over the five-week period are presented in Table 3. The TH was highest at point Cp with 1.79 × 10^4^ cfu/mL and lowest at point Dp with 1.09 × 10^4^ cfu/mL (Table 2). The TH varied within the weeks as it oscillated between 4.70 × 10^3^ cfu/mL in week 5 and 1.84 × 10^4^ cfu/mL in week 2 (Table 3). The total heterotrophic count (TH) obtained in week 5 was significantly low and statistically different (*p <* 0.05) from the values obtained in the first four weeks. (Table 3).The total coliform count (TC) was relatively high and temporally varied as the values oscillated from 2.32 × 10^3^ cfu/100mL in week 5 to 6.20 × 10^3^ cfu/100mL in week 3 (Table 3). Total coliform count varied spatially and was generally high as it ranged from 3.56 × 10^3^ cfu/100mL to 5.24 × 10^3^ cfu/100mL. An alarming high mean TC count was obtained in point Cp (Table 2). The total thermotolerant coliform count (TTC) ranged from 1.24 × 10^3^ cfu/100mL in week 5 to 4.80 × 10^3^ cfu/100mL in week 3. The highest TTC along different sampling points was also recorded at point Cp with 3.22 × 10^3^ cfu/100mL as observed with the total coliform count of the same point. The TTC values were all relatively higher than the recommended limit for river water quality [26].

The percentage and ratio of mean total thermotolerant coliform count (TTC) to mean total coliform count (TC) was presented in Table 4. The TTC: TC percentages were all above 50% showing that more than half of the total coliforms are thermotolerant or are of faecal origin. The results of the physicochemical and bacteriological analysis of the Asa Dam water (control site) conformed to the limits of the WHO recommended regulatory standard for aquatic environments (data not shown).

**Figure 5:**
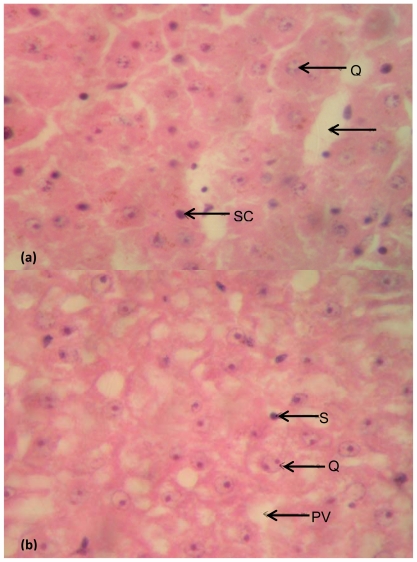
(**a**) Histological structures of the liver of *Clarias gariepinus* caught from Asa River segment; (**b**) Histological structures of the liver of *Clarias gariepinus* caught from Asa Dam water (Control). Plates showed no distinct histological damage in the liver of the test fish compared to the control (at magnification 100×). Arrows showed the binucleated hepatocytes (Q), the clear portal venules (PV) and the sinusoidal lining cells (SC).

**Figure 6:**
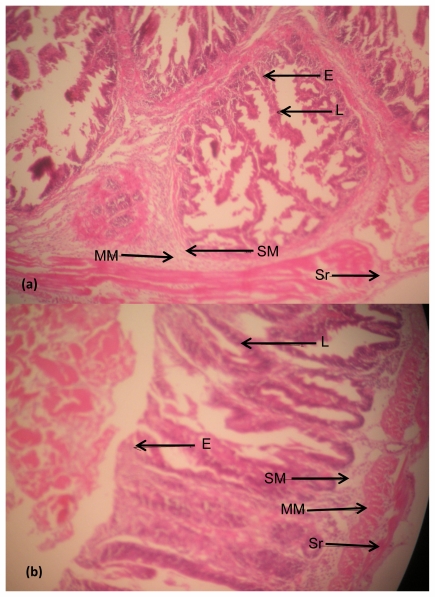
(**a**) Histological structures of the intestine of *Clarias gariepinus* caught from Asa River segment; (**b**) Histological structures of the intestine of *Clarias gariepinus* caught from Asa Dam water (control). Plates showed no distinct histological damage in the intestine of test fish compared to the control (at magnification 100×). Arrows showed the distinct layers of the intestinal Mucosa (Epithelium, E and lamina propia, L), the Submucosa (SM), the Muscularis (MM) and the Serosa (Sr).

**Figure 7:**
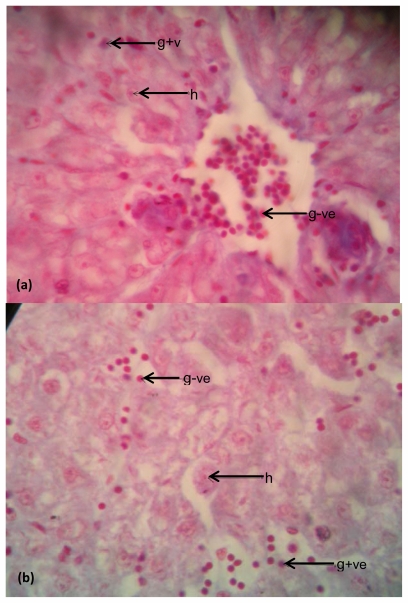
(**a**) Examination of bacteria in liver tissues of *Clarias gariepinus* obtained from Asa River segment; (**b**) Examination of Bacteria in liver tissues of *Clarias gariepinus* obtained from Asa Dam water. Plates showed a higher bacterial density in the liver tissues of the test fish than in the control (at magnification 400×). Arrows showed the Gram-negative bacteria (shades of red), few Gram-positive bacteria (blue-black shades) and hepatocytes (h) on the photomicrographs.

**Figure 8:**
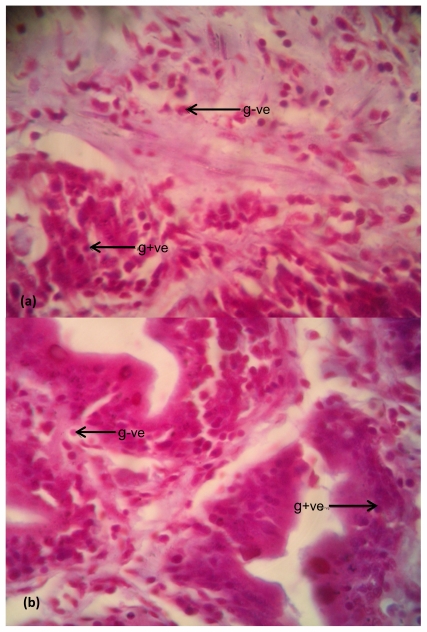
(**a**) Examination of bacteria in the intestinal tracts of *Clarias gariepinus* obtained from Asa River segment; (**b**) Examination of Bacteria in the intestinal tracts of *Clarias gariepinus* obtained from Asa Dam water. Plates showed a small increase in the bacterial density in the intestine of test fish compared to the control (at magnification 400×). Arrows showed the numerous Gram-negative bacteria (in red shades (g−ve) and few Gram-positive bacteria [blue-black shades (g+ve)] on the photomicrographs.

Photomicrographs of representative slides showing histological structures of stained tissue sections of the fish liver and intestine are presented in Figures 5 and 6, while the photomicrographs showing the differentiated bacterial population present in the liver and intestinal tissues are presented in Figures 7 and 8.

### Liver

The histological sections of the liver tissues structures of *Clarias gariepinus* from Asa River and Asa Dam water (control site) as shown in Figure 5(a,b) respectively. The hepatocytes of both sections are normal. They are mostly binucleated and multinucleated. They are normochromic at higher magnification (400×). The nucleoli are clearly seen and there are few areas of sinusoids containing erythrocytes. The fish liver tissues from the Asa River and Asa Dam water (control site) were comparatively normal with no visible alterations.

### Intestine

The histological sections of the intestine tissues structures of *Clarias gariepinus* from Asa River and Asa Dam water (control site) as shown in Figure 6(a,b), respectively. The digestive tract sections were well stained with distinct normal layers from mucosa to the serosa. The mucosa (columnar epithelium and lamina propia), submucosa (loose connective tissue), muscularis (circularis and longitudinale) and serosa (mesothelium) are clearly seen with higher magnification (100×). Slides of Figure 6(a,b) are from the pyloric caeca. Comparatively, they are the same with no visible alterations.

### Bacteria in Liver

The photomicrographs of the differentiated bacterial cells present in the liver tissues of *Clarias gariepinus* from the test Asa River and the control Dam water as shown in Figure 7(a,b), respectively. Blue-black shades of cells denote the Gram-positive bacteria while shades of red colour denote Gram-negative bacteria and hepatocytes. Figure 7(a) shows numerous Gram-positive and Gram-negative cells which are concentrated and dispersed in some areas within the tissue section. The Gram-negative cells are more densely populated in the liver than the Gram-positive cells as indicated by more red shades on the plate. Figure 7(b) reveals purely dispersed but scanty Gram-negative and Gram-positive cells. However, Figure 7(a) indicates a comparatively higher microbial load/density in the liver than that seen in Figure 7(b).

### Bacteria in Intestine

The differentiated bacteria cells present in the intestinal tissue sections of *Clarias gariepinus* from the test Asa River and the control Asa Dam water as shown in Figure 8(a,b), respectively. There was a small distinction with a little increase in the bacterial population in the intestine of *Clarias gariepinus* from Asa River over the bacterial population in the intestine of *Clarias gariepinus* from the Asa Dam water. Figure 8(a) shows concentrated Gram-negative and Gram-positive bacteria in the digestive tract. Figure 8(b) also shows concentrated Gram-negative cells but scanty gram-positive cells.

## 4. Discussion

Water quality is neither a static condition of a system, nor can it be defined by the measurement of only one parameter. There is a range of chemical, physical and biological components that affect water quality. These variables provide general indication of water pollution, whereas others enable a direct tracking of pollution sources [1].

The pH of water samples collected in time and space from the river section were slightly below neutral (Table 1 and Figure 2) and these values fall within the accepted range of 6.0–8.5 indicative of good water quality [1,27,28]. The mean temperature values of the water samples are not statistically different from each other (*p* < 0.05) and also fall within the normal temperature range supportive of good surface water quality which is 0 °C to 30 °C [28]. Hence, the temperature of the water from Asa River could not be implicated as influencing the observed variations in the bacterial population as well as in other physicochemical parameters. However, the observed range of the temperature allows for optimum proliferation of most of the bacteria isolated from the water samples. Enterobacteriaceae are mesophiles most of which grows optimally at temperature range of 20 °C and 32 °C [29].

The significantly high total suspended solids (TSS) and total dissolved solids (TDS) of the water (*p* < 0.05) are implicative of a high level of pollution of the Asa River when compared to the WHO standard limit for good water quality which is 500 mg/L for TSS and 1,000 mg/L for TDS [28]. TSS and TDS are indicative of materials carried in suspension and solid respectively [30]. Suspended solids in streams are often as a result of sediments carried by the water whose source includes natural and anthropogenic (human) activities in the water shed, such as natural or excessive soil erosion from agriculture, forestry or construction, urban run-off, industrial effluents or phytoplankton growth [31]. Suspended solids content was also reported to be directly proportional to instantaneous discharge of effluents measured in the Yellow River in China [1]. The high TSS and TDS content values of the water show significant direct relationships to the high bacterial population obtained from the water samples.

The significant decrease in the TSS and TDS content of water samples collected at point Cp, the point which tends downwards and northwards away from points Ap and Bp could be linked to the observed correspondingly lowest count of the total heterotrophic bacteria enumerated at the same point Cp of interest. This observed decrease could be linked to the effect of self-purification process at the flowing stream which could reduce the microbial population.

The high BOD and very high COD values are indicative of the presence of organic and inorganic pollutants, respectively. The mean BOD values of all exceeded the recommended maximum allowable concentration (RMC) set by the European Union for good quality water for fisheries and other aquatic life which is 3.0–6.0 mg/L [28]. Unpolluted waters typically have BOD values of 2 mg/L or less, whereas those receiving wastewaters may have values up to 10 mg/L or more [28]. It was reported that these parameters *i.e.*, BOD and COD are responsible for odour and taste [32]. The COD usually includes all, or most of the BOD as well as some other chemical demands. The significantly high mean COD values also exceeded the acceptable concentrations for unpolluted surface water quality which is 20 mg/L or less. It however falls within the range of polluted waters (20–200 mg/L) [28].

The high BOD and COD values may be due to the discharge of untreated or incompletely treated industrial effluents into the river from the various manufacturing plants in the industrial estate such as Global Soaps and Detergents, Seven-Up Bottling Company, Nigeria Bottling Company (Coca-Cola), Dangote Flour Mills, Rajrab Pharmaceutical Company, *etc*. The BOD values are high probably due to discharge of domestic wastes especially defecation activities and poorly executed agricultural activities near the river banks which was observed during survey of study site. The downward trend in the BOD values along the sampling points Ap to Dp (Table 1) which goes in a northward direction with the river flow indicates a decrease in the organic pollution level. This might be due to the self-purification process in the river. The self-purification process in a river is a phenomenon that has been noted in waters contaminated by sewage. It is a complex process running in many stages [33–35].

A faecal coliform index obtained by a ratio of faecal coliforms to total coliforms, has been proposed and recommended by USEPA instead of the formally used total coliform index for evaluating the microbiological suitability of freshwaters for recreational uses. The proposed maximum acceptable limit was 200 faecal coliforms per 100 mL and 126 *E. coli* per 100 mL [26]. This preference was because faecal coliforms were more faecal-specific and less subject to variation than total coliforms which were greatly influenced by storm water run-off. It has also been found that, usually, more than 95 percent of thermotolerant coliforms isolated from water are the gut organism *Escherichia coli*, the presence of which is definitive proof of faecal contamination [36].

The alarming high number of total coliforms and thermotolerant (faecal) colifoms per 100 mL obtained from the water samples, which exceeds at least ten times the recommended limit, indicates high level of faecal pollution of the river water which potentially poses a high health risk for recreational purposes, let alone for drinking purpose. This clearly implies that the organic pollution of the river is more of faecal pollution. It was reported in a previous study on the Ohio River at Dayton in the US that swimmers who swam in water with a median coliform density of 2,300 coliforms per 100 mL had an excess of gastrointestinal illness rate when compared to an expected rate calculated from the total study population [26]. It was posited that the total number of heterotrophic bacteria reflects the contamination extent by the easily decomposable organic matters, while the faecal coliform bacteria number gives an idea of the contamination size by faecal substance [37].

The ANOVA test showed that there are significant variations in the total coliform counts and the thermotolerant coliforms counts between weeks on all sampling points (*p* < 0.05). The variations might be due to climatic factors such as rainfall and changes in the anthropogenic activities during the period of analysis. The occurrence of irregular variations in coliform bacteria was congruent with the findings of [10] and [38]. In most circumstances, concentrations of the thermotolerant coliforms are directly related to that of *E. coli*. The presence of *E. coli* or thermotolerant coliform bacteria gives an indication of presumptive presence of water-borne pathogens [11,39].

The histological findings on the tissue structures of the four different parts of *Clarias gariepinus* collected from experimental Asa River section depicted no significant alterations or pathological effects in the livers and intestines when compared with those collected from the control Asa Dam water. This result does not corroborate with similar studies on fish species of *Tilapia zillii* and *Solea vulgaris* from Lake Qarun in Egypt which observed histological alterations in the muscles including vacuolar degeneration and aggregation of necrosis in gills and inflammatory cells in hepatocytes of liver tissues [6].

The observed histological alterations in the fishes studied in Lake Qarun was possible simply because the lake is a closed system which acts as a reservoir for agricultural and sewage drainage water and whose components are found to be polluted with heavy metals [40,41]. However, such alterations could not be found in the viscera of the studied fish species from Asa River being inhabitants of a flowing system and owing to the possibilities of pollution dilution and self-purification in the river. This would possibly reduce the bioaccumulation rate of the water pollutants in the tissues of the resident fish.

Previous toxicological evaluation studies on the Amilegbe segment of Asa River by haematological and enzyme studies of rats fed the polluted river water revealed marked haematological changes and effects on enzymes of the rat after a period of eight weeks [30]. This was achievable as it is a controlled environment outside the field. The results of the present study has not proven the histopathology of live indigenous fishes as an effective biomarker of *in situ* river pollution impact of non-point source pollution, though it has been for chronic exposure studies on specific metal concentrations [42–46].

Nevertheless, the high microbial load observed on the gram-stained histological slides of the fish liver and intestine (Figures 7 and 8) is in agreement with the results of the physicochemical and bacteriological evaluation of Asa River pollution and its impact on the aquatic life. There are comparative distinctions. The dense bacterial population observed in the fish intestine of both Asa River and the control water could be due to the enteric region of the fish. The increase in microbial load observed in the fish liver from Asa River when compared to the control reveals pollution effect in the fishes of the river.

The comparative study done between the catfish of the Asa Dam reservoir water (control) and the polluted Asa River segment revealed, by bacterial examination in the fish tissues observed through special staining, a comparatively significant alteration of the normal microbial flora of the catfish from the polluted river.

In his work, Strauss reported that invasion of fish flesh by pathogenic bacteria was very likely if the fish were reared in water containing over 10^4^ of faecal coliforms (*Escherichia coli*) and that high concentrations of pathogenic microorganisms might occur in the digestive tract and intraperitoneal fluid of the fish even at low numbers of indicatory bacteria [47]. The values pointed out in Strauss’ work were within range with the values obtained in the bacteriological examination of Asa River water. These findings, however, render the microbiological quality of the fishes from the polluted river poor and unsafe for consumption.

## 5. Conclusions

This pollution impact study has proven that the downstream Asa River segment is indeed polluted. The study has been able to track the type of pollution to be more of faecal contamination by the evaluation of the bacteriological quality of the river water samples. Non-point sources of pollution which include the agricultural activities (pesticides and crop wastes) and domestic activities by the poorly planned settlers nearby the river as well as the point source discharge of industrial effluents along the industrial estate have been implicated to be causative to the poor quality of the river and its aquatic life.

This study has also shown that there were no significant tissue alterations observed during the comparative histological studies carried out on juvenile live samples of *Clarias gariepinus* caught from the polluted Asa River and the control Asa Dam water. However, this does not imply good quality of the aquatic life of the river as further investigation of the bacteria present in the fish tissues portrayed very high bacterial density in the tissues of *Clarias gariepinus* collected from the Asa River segment when compared to the tissues of *Clarias gariepinus* caught from the Asa Dam water. The implications of these findings may be that people dependent on this river water for domestic use including cooking, bathing, washing and even drinking or for agricultural uses like fishing and farming may be exposed to public health risks. Proper treatment is imperative for the river to be appropriate for potable, domestic and industrial purposes.

It is therefore recommended to coordinate different efforts at the level of the community dwellers and the government to rescue the downstream Asa River segment and its aquatic life from the current hazard-posing environmental problems.

## Figures and Tables

**Table 1:** Physicochemical properties of water samples taken at different points from Asa River Segment.

Parameter measured	Sampling Points			

	A_p_ (point 1)	B_p_ (point 2)	C_p_ (point 3)	D_p_ (point 4)
**pH**	6.43 ± 0.25 ^a^	6.32 ± 0.28^a^	6.33 ± 0.30 ^a^	6.32 ± 0.26 ^a^
**Temp. (°C)**	25.20 ± 0.56 ^a^	24.50 ± 0.22 ^a^	24.90 ± 0.46 ^a^	25.30 ± 0.25 ^a^
**TSS (mg/L)**	1,163.40 ± 242.23 ^b^	993.40 ± 267.81 ^b^	766.60 ± 347.47 ^b^	1,498.60 ± 627.78 ^b^
**TDS (mg/L)**	1,201.40 ± 308.14 ^b^	1,799.80 ± 1072.71 ^b^	704.60 ± 231.53 ^b^	754.60 ± 198.71^a^
**COD (mg/L)**	33.60 ± 7.78 ^a^	58.40 ± 13.21^a^	27.20 ± 5.12 ^a^	23.20 ± 5.43^a^
**BOD (mg/L)**	14.00 ± 7.15 ^a^	12.12 ± 2.68^a^	11.28 ± 2.38 ^a^	6.48 ± 2.10 ^a^

Values are presented as Mean *±* SEM (n = 5).

All groups are compared to each other at P < 0.05. Values with different superscripts along the same column are statistically different from each other.

**Table 2:** Mean Total Heterotrophic, Total Coliform and Total Thermotolerant Coliform counts of the water samples from four sampling points on Asa River Segment.

Sampling Points	Total Heterotrophic count (TH) (cfu/mL)	Total Coliform count (TC) (cfu/100mL)	Total Thermotolerant Coliform count (TTC) (cfu/100mL)	Freshwater Quality Standard Limit (Faecal coliforms per 100 mL) *
***A****_p_****(point 1)***	1.24 × 10^4^ ± 2,891.27 ^a^	3.56 × 10^3^ ± 844.16 ^a^	2.99 × 10^3^ ± 767.03 ^a^	200
***B****_p_****(point 2)***	1.24 × 10^4^ ± 3,414.67 ^a^	4.50 × 10^3^ ± 804.36 ^a^	2.49 × 10^3^ ± 641.57 ^a^	200
***C****_p_****(point 3)***	1.79 × 10^4^ ± 6,615.26 ^a^	5.24 × 10^3^ ± 1,088.39 ^a^	3.22 × 10^3^ ± 701.00 ^a^	200
***D****_p_****(point 4)***	1.09 × 10^4^ ± 2,309.46 ^a^	3.84 × 10^3^ ± 946.89 ^a^	2.88 × 10^3^ ± 876.58 ^a^	200

* Source: USEPA [26].

Values are presented as Mean ± SEM (n = 5).

All groups are compared to each other at P < 0.05. Values with the same superscripts along the same column are not statistically different from each other.

**Table 3:** Mean Total Heterotrophic, Total Coliform and Total Thermotolerant Coliform counts of the water samples taken for a five-week period from Asa River.

Sampling Period	Total Heterotrophic count (TH) (cfu/mL)	Total Coliform count (TC) (cfu/100mL)	Total Thermotolerant Coliform count (TTC) (cfu/100mL)	Freshwater Quality Standard Limit (Faecal coliforms per 100 mL) *
***Week 1***	1.12 × 10^4^ ± 2,015.564 ^ab^	3.77 × 10^3^ ± 311.92 ^a^	2.67 × 10^3^ ± 661.28 ^ab^	200
***Week 2***	1.84 × 10^4^ ± 3084.47 ^b^	6.07 × 10^3^ ± 878.80 ^b^	3.80 × 10^3^ ± 435.89 ^bc^	200
***Week 3***	1.50 × 10^4^ ± 408.25 ^ab^	6.20 × 10^3^ ± 906.46 ^b^	4.80 × 10^3^ ± 624.50 ^c^	200
***Week 4***	1.77 × 10^4^ ± 8,410.06 ^ab^	3.05 × 10^3^ ± 723.99 ^a^	1.96 × 10^3^ ± 366.59 ^a^	200
***Week 5***	4.70 × 10^3^ ± 624.50 ^a^	2.32 × 10^3^ ± 232.29 ^a^	1.24 × 10^3^ ± 189.80 ^a^	200

* Source: USEPA [26].

Values are presented as Mean ± SEM (n = 4).

All groups are compared to each other at P < 0.05. Values with different superscripts along the same column are statistically different from each other.

**Table 4:** Percentage and ratio of thermotolerant (faecal) coliforms to total coliforms (TTC:TC) of water samples over a period of 5 weeks.

Sampling Period	Mean total coliform counts (TC) (cfu/100mL)	Mean total thermotolerant coliform counts (TTC) (cfu/100mL)	Percentage TTC in TC (%)	Faecal Coliform Index (TTC: TC Ratio)
**Week 1**	3.77 × 10^3^	2.67 × 10^3^	70.86	0.71
**Week 2**	6.07 × 10^3^	3.80 × 10^3^	62.55	0.63
**Week 3**	6.20 × 10^3^	4.80 × 10^3^	77.42	0.77
**Week 4**	3.05 × 10^3^	1.96 × 10^3^	64.51	0.65
**Week 5**	2.32 × 10^3^	1.24 × 10^3^	53.55	0.54
